# Expected changes in obesity after reformulation to reduce added sugars in beverages: A modeling study

**DOI:** 10.1371/journal.pmed.1002664

**Published:** 2018-10-05

**Authors:** Ana Basto-Abreu, Ariela Braverman-Bronstein, Dalia Camacho-García-Formentí, Rodrigo Zepeda-Tello, Barry M. Popkin, Juan Rivera-Dommarco, Mauricio Hernández-Ávila, Tonatiuh Barrientos-Gutiérrez

**Affiliations:** 1 National Institute of Public Health, Center for Population Health Research, Cuernavaca, Mexico; 2 Department of Nutrition, University of North Carolina Gillings School of Global Public Health, Carolina Population Center, Chapel Hill, North Carolina, United States of America; 3 National Institute of Public Health, Cuernavaca, Mexico; 4 University Center of Los Altos, Tepatitlan de Morelos, University of Guadalajara, Guadalajara, Jalisco, Mexico; Centers for Disease Control and Prevention, UNITED STATES

## Abstract

**Background:**

Several strategies have been proposed to reduce the intake of added sugars in the population. In Mexico, a 10% sugar-sweetened beverages (SSBs) tax was implemented in 2014, and the implementation of other nutritional policies, such as product reformulation to reduce added sugars, is under discussion. WHO recommends that all individuals consume less than 10% of their total energy intake (TEI) from added sugars. We propose gradually reducing added sugars in SSBs to achieve an average 10% consumption of added sugars in the Mexican population over 10 years and to estimate the expected impact of reformulation in adult body weight and obesity.

**Methods and findings:**

Baseline consumption for added sugars and SSBs, sex, age, socioeconomic status (SES), height, and weight for Mexican adults were obtained from the 2012 Mexico National Health and Nutrition Survey (ENSANUT). On average, 12.6% of the TEI was contributed by added sugars; we defined a 50% reduction in added sugars in SSBs over 10 years as a reformulation target. Using a dynamic weight change model, sugar reductions were translated into individual expected changes in body weight assuming a 43% caloric compensation and a 2-year lag for the full effect of reformulation to occur. Results were stratified by sex, age, and SES. Twelve years after reformulation, the TEI from added sugars is expected to decrease to 10%, assuming no compensation from added sugars; 44% of the population would still be above WHO recommendations, requiring further sugar reductions to food. Body weight could be reduced by 1.3 kg (95% CI −1.4 to −1.2) in the adult population, and obesity could decrease 3.9 percentage points (pp; −12.5% relative to baseline). Our sensitivity analyses suggest that the impact of the intervention could vary from 0.12 kg after 6 months to 1.52 kg in the long term.

**Conclusions:**

Reformulation to reduce added sugars in SSBs could produce large reductions in sugar consumption and obesity in the Mexican adult population. This study is limited by the use of a single dietary recall and by data collected in all seasons except summer; still, these limitations should lead to conservative estimates of the reformulation effect. Reformulation success could depend on government enforcement and industry and consumer response, for which further research and evidence are needed.

## Abstract 2

Tonatiuh Barrientos-Gutiérrez and colleagues use a weight reduction model to estimate individual level weight reductions 12 years after the introduction of the 2012 Mexican sugar tax.

## Author summary

**Why was this study done?:**

- High sugar intake is associated with obesity.
- Adults in Mexico consume 12.6% of their everyday energy intake from added sugars, exceeding WHO recommendations (<10% of total energy intake [TEI]).
- The Mexican government imposed a 10% sugar-sweetened beverages (SSBs) tax to reduce sugar consumption; other interventions such as reformulation and gradual elimination of sugar are being discussed.

**What did the researchers do and find?:**

- We estimated the proportion of added sugars that should be reduced in SSBs to reduce sugar intake to less than 10% of everyday energy intake. We found that we could achieve this goal by halving the sugar content in SSBs.
- Using mathematical models, we translated the reductions in sugar consumption into expected changes in body weight and the prevalence of obesity in adults.
- Allowing for 43% caloric compensation, we estimated that 12 years after starting the sugar reduction in SSBs, the Mexican population would experience an average reduction of 1.3 kg and 12.5% less obesity.
- Larger effects of the sugar regulation will be expected among males, young adults, and the middle socioeconomic status (SES) group.

**What do these findings mean?:**

- Our study shows that a national regulation to reduce added sugars in SSBs could be an effective intervention to reduce sugar intake and curb the obesity epidemic in Mexico.
- The sugar regulation should be considered as a part of an integral strategy by the Mexican government to reduce obesity and avert obesity-related diseases.

## Introduction

High sugar intake is a primary risk factor for obesity. Sugar intake has been associated with weight gain [1,2], diabetes, and other diseases [3–5]. Recent calls have been made to reduce sugar intake, in particular targeting sugar-sweetened beverages (SSBs) [6,7]. SSBs provide 39% of daily sugar intake in the United Kingdom [6] and 60% in the United States [8]. In 2011, Mexico was the largest SSB consumer worldwide, with an average of 163 liters per capita [9]; SSBs provided 69% of total added sugar in the population’s diet [10]. Adults in Mexico consume 12.6% of their total energy intake (TEI) from added sugars, exceeding the intake recommended by US dietary guidelines (<10% TEI from added sugars) and WHO (<10% TEI from free sugars) [7,11].

A first step towards regulating SSB consumption was taken in January 2014 by the Mexican Government when it imposed a 10% tax on all industrialized SSBs, which produced a 9.7% purchase reduction by 2015 [12]. Still, more interventions are needed to further decrease sugar consumption and produce larger health benefits [13]. In recent years, a movement towards defining maximum concentrations of specific substances (with sodium intake being most prominent) in the food industry has gained momentum [14–16]. Countries have also used more complex grams of sugar and tiered taxation schemes to foster sugar reformulation [17]. For instance, in 2016, the UK government challenged the industry to reduce added sugars in products consumed by children, leading to an 11% reduction in the sugar concentration of SSBs [18]. In this context, setting a maximum limit to added sugars in SSBs is a reasonable intervention to decrease sugar intake and obesity.

Mexico is an ideal site to analyze the potential implementation of a national regulation to decrease added sugar content in beverages. The health secretary has declared obesity and diabetes public health emergencies, calling for decisive actions to curb these epidemics [19], and the National Strategy to Control and Prevent Overweight, Obesity and Diabetes considers the reformulation of beverages and food as well as encourages the gradual elimination of sugar, sodium, and saturated fats [20]. We aimed to estimate the proportion of sugar that should be reduced in SSBs to reduce added sugar intake in the population to an average 10% TEI, assuming no changes in consumption. Using a representative sample of adults, we estimated the expected impact of a sugar regulation of SSBs on weight and prevalence in obesity and overweight over 12 years.

## Methods

### Data collection

We obtained baseline SSB consumption, weight, and prevalence of overweight and obesity among adults in Mexico using the 2012 National Health and Nutrition Survey (ENSANUT). The ENSANUT 2012 is a cross-sectional, multistage, probabilistic survey representative of the Mexican population that measures the health and nutrition status in the Mexican population. The ENSANUT 2012 surveys 50,528 households, with a total of 96,031 individuals, including children, adolescents, and adults. This survey includes demographic, socioeconomic, nutritional, and health-related data. Nutritional information was collected on a representative subsample of 10,886 individuals (approximately 11%). The National Institute of Public Health estimated the individual sampling weights in ENSANUT needed to represent the age and sex distribution of the Mexican population and to account for survey nonresponse [21].

To collect baseline information from dietary intake and body mass index (BMI), we used the food 24-hour recall and anthropometry databases, including adults 20 years of age and older. Extreme values of energy intake ($\text{more}\;\text{then}\;\pm 3\;SDs\;ln\left(\frac{total\;energy\;intake}{total\;energy\;requirements}\right)$, *n* = 125) [22], extreme values of SSB consumption (>3,000 kcal; *n* = 1), children <1 year, breastfeeding children, and pregnant and lactating women were excluded (*n* = 665) because energy intake varies during these periods. Participants younger than 20 years (*n* = 6,926) and those with missing data for weight and height (*n* = 164) were excluded from the analysis.

### Dietary intake

We collected added sugar consumption (from SSBs and other products) using the 24-hour diet recall from ENSANUT for the adult population. SSBs included all industrialized nonalcoholic beverages taxed in 2014 in Mexico (a list of beverages is available in section 1.1 of Supporting Information S1 Methods). The food composition table compiled to analyze ENSANUT’s 24-hour diet recall does not include added sugar. This nutrient was previously estimated by Sánchez-Pimienta and colleagues [10]. Briefly, added sugar was set to zero for all foods without sugar or foods for which all sugar is intrinsic (such as fruits, unprocessed cereals, or legumes); added sugar was set to the total sugar content for foods for which all sugar is added (such as sodas, confectionary, and sweeteners). Added sugar was estimated as a share of the total sugar content for all other foods having a mix of intrinsic and added sugar. This share was estimated based on either lactose content or similar foods that had no added sugars, for instance, comparing the sugar content from 100% juice versus industrialized juice (understanding industrialized juices as beverages that contain some proportion of fruit juices but with added ingredients such as sugar, additives, or conservatives).

### BMI

Weight and height were directly measured by trained interviewers during home visits using standardized examination procedures [21]. BMI was calculated using weight (kg) divided by squared height (m^2^). We classified each adult following WHO guidelines, but given the low prevalence of underweight (0.8% with <18.5 kg/m^2^), we collapsed this category with the normal range (BMI ≥ 18.5 and < 25 kg/m^2^); overweight (BMI ≥ 25 and < 30 kg/m^2^) and obesity (BMI ≥ 30 kg/m^2^) were reported as usual.

### Stratification variables

SSB consumption varies according to sex, age, and socioeconomic status (SES) [23]. Continuous age was introduced in the weight change model, yet we categorized it into young adults 20 to 40 years old, adults 40 to 60 years old, and elderly (age 60 and older) to facilitate the presentation of results. SES was constructed for the ENSANUT 2012 using demographic and socioeconomic characteristics of the household head (years of education, sex, and employment status), sociodemographic structure (individuals living in the household, individuals employed, adults employed, children working, and index of economic dependence), household characteristics (number of rooms, exclusive kitchen, bathroom, and type of fuel, among others), household assets (TV, microwave, and computer, among others), family consumption patterns (housing rent and logarithm of household expenditures), and characteristics of the place of residence using the 2010 marginalization index from the National Council on Population, which considers education, housing, and income at the local level [24]. For this analysis, we used three categories of SES (low, middle, and high), which represent SES tertiles of the adult population in the anthropometric ENSANUT database.

### Reduction of the amount of added sugar

To estimate the amount of added sugar that should be reduced in SSBs to reduce the population intake to less than 10% TEI, we calculated the total added sugar consumption from all dietary sources for each ENSANUT participant. At baseline, adults in Mexico consumed on average 12.6% of TEI from added sugar, exceeding WHO and US guideline recommendations [7,11]. While the WHO recommendation is for all individuals, we estimated the sugar reduction required to achieve an average sugar consumption of 10% at the population level, which implies that 44% of individuals would still be above the recommended 10% TEI (assuming no compensation for added sugars).

To calculate the average reduction in sugar from SSBs required to reach the average 10% TEI goal, we first estimated the individual maximum added sugar consumption from SSBs, as follows (Eq 1):

$$10\%\;=\;\frac{{SSB}_{max}^{i}+{others}^{i}}{{TEI}_{init}^{i}+({SSB}_{max}^{i}-{SSB}_{init}^{i})},$$ (1)

where “others” is the consumption of added sugars from sources other than SSBs, *TEl*_*init*_ is the TEI at baseline, and *SSB*_*init*_ is the added sugars from SSBs. If current consumption from SSBs was lower than the maximum calculated (*SSB*_*init*_ < *SSB*_*max*_), no changes were made Δ_*change*_ = 0; if *SSB*_*init*_ < *SSB*_*max*_, the change in added sugars was Δ_*change*_ = *SSB*_*init*_ − *SSB*_*max*_. If added sugar consumption from other sources was above 10% TEI, then we reduced all sugar from SSBs so that Δ_*change*_ = −*SSB*_*init*_. The average proportion of added sugars in SSBs was calculated based on the individual change, $\sum_{i=1}^{N}\omega_{i}\cdot prop_{i}=\sum_{i=1}^{N}\omega_{i}\cdot \frac{{}_{{}^{\Delta }change^{i}}}{SSB_{init}{}^{i}}=52.2\%$, which we rounded down to 50%. A detailed description of how we estimated the target reduction in added sugars can be found in Supporting Information S1 Methods, section 2.1 and 2.2.

To halve the sugar content, we proposed a national regulation to gradually reduce added sugars in SSBs to reach the goal over a 10-year period. We set a long implementation period with small yearly sugar reductions to allow for a gradual adaption to the new level of sweetness. The first-year reduction was estimated as follows (Eq 2):

$$Reduction{(y}_{1})\;=\;{1-(1-Reduction(y_{k}))}^{1/k}$$ (2)

where *k* denotes the number of years the regulation takes place. Considering *Reduction* (*y*_10_) = 0.5 = 50%. We obtained a first-year reduction of *Reduction* (*y*_1_) 0.067 = 6.7%. The accumulated reductions in the following years were estimated as follows (Eq 3):

$$Reduction\;{(y}_{k})\;=\;1-(1-{Reduction(y_{1}))}^{k},$$ (3)

where *Reduction* (*y*_*k*_) is the estimated reduction for each year *k* = 1, 2,…,10. Hence, the reduction at *y*_2_ would be 12.9%, at *y*_3_ would be 18.8%, and so on to achieve a 50% reduction by *y*_10_. A detailed description of the yearly reduction estimation can be found in Supporting Information S1 Methods (section 2.3).

### Change in energy intake

To estimate the daily change in caloric intake, *ΔCaloric intake* attributable to the sugar reduction in SSBs, we multiplied the individual added sugar intake from SSBs by the cumulative percent reduction in sugar content for that year considering compensation, as follows (Eq 4):

$$\Delta Caloric\;intake(y_{k})\;=\;Added\;sugar\;SSBs\;\times Reduction(y_{k})\times (1-\frac{Compensation}{100}).$$ (4)

Compensation is understood as the adjustments in energy intake that follow to the reduction induced by the intervention (in this case, the reduction in added sugars in SSBs). We assumed a 43% compensation based on a recent meta-analysis that analyzed the substitution of added sugars by low-caloric artificial sweeteners [25]. Under 43% compensation, an individual reducing 50 kcal/day would compensate 21.5 kcal through sources other than added sugars, resulting in a net reduction of 28.5 kcal/day. The average change in energy intake was stratified by sex, age group, and SES. Aggregated changes in energy intake were estimated using Stata/MP version 14.1 [26] taking into account the complex survey design (see Supporting Information S1 Methods, section 1.2).

### Change in body weight and obesity prevalence

The change in body weight was estimated using the dynamic weight change model proposed by Chow and Hall [27], implemented individual by individual for all participants in the ENSANUT. This model accounts for the dynamic physiological adaptation that occurs following weight loss, and it has been validated with experimental data [27–29]. Briefly, the model uses a system of differential equations to predict the expected body weight change of an individual. It is estimated as the sum of extracellular fluid, glycogen, fat mass, and lean tissue, dependent on sodium and energy intake, energy expenditure, and other characteristics such as sex, age, height, initial body weight, and physical activity level [27]. Our model assumes steady-state for body weight and no changes in physical activity nor in energy intake (apart from the sugar regulation) during the modeling period. A step-by-step explanation of the implementation of the model is provided in S1 Methods, section 3.

Using the expected reduction in energy intake, we implemented Chow and Hall’s model to each adult in ENSANUT 2012 to obtain the expected reduction in weight after 12 years (considering a 2-year time lag for caloric changes to influence weight). Final weights were transformed to BMI and BMI categories (normal, overweight, obese) to estimate prevalence changes. Assuming that the sugar regulation would be implemented in 2020, we estimated the number of prevented overweight and obesity cases by 2032 by multiplying the change in prevalence in percentage points (pp) by the expected adult population in 2032, using population projections published by the National Population Council (CONAPO) [30]. The dynamic body weight model was programmed using R statistical software version 3.4.1 (2017-06-30) [31] using the bw package [31] and the Rcpp package [32–35]. Aggregated changes in body weight and BMI status by sex, age group, and SES were estimated using Stata/MP 14.1 [26], taking into account the complex survey design (see Supporting Information S1 Methods, section 1.2). The final dataset used is available at https://osf.io/vfcm8/ (doi: 10.17605/OSF.IO/VFCM8), and the variables are explained in S1 Methods (section 1).

### Sensitivity analyses

In the main scenario, we assumed 43% energy compensation based on a recent meta-analysis [25]. This was a request from the peer review process. In our first analysis, we had assumed no energy compensation given the discordant heterogeneity of results included in the meta-analysis and other sources suggesting minimal or zero dietary compensation for liquids [36–38]. Results with 0% compensation were included as a sensitivity analysis (Table A in S1 Results). We also conducted a sensitivity analysis to estimate all possible compensation scenarios (from 0% to 100%) and different added sugar reduction targets (from 0% to 100%) in a 10-year period following the “decreasing yearly reduction” (Eq 3). The overall reduction for each added sugar target and each compensation level was estimated as follows (Eq 5):

$$Reduction(y_{1})\;=\;Target\;\times (1\;-\frac{Compensation}{100}).$$ (5)

where *Target* represents the maximum reduction achieved with the regulation. The change in energy intake was estimated using Eq 4 and applied it to the individual weight change model. For additional information, including a matrix summarizing weight reductions, see Supporting Information S1 Methods (section 4).

For the main analysis, the regulation set a 6.7% added sugar reduction in the first year, followed by lower percent reductions for the subsequent years. We called this scenario “decreasing yearly reduction.” Two other implementation scenarios were estimated with a 50% reduction of added sugar gradually achieved over 10 years, yet we decided to present results after 12 years because, at that point, the three scenarios converged. The “constant yearly reduction” scenario reduces added sugar by 5% yearly from the baseline content. The “increasing yearly reduction” scenario considers the same percent changes as the “decreasing yearly reduction” but in reverse order: starting with a small decrease (y_1_ = 3.6%) and augmenting the percent reduction (y_10_ = 6.7%) to reach 50% by year 10. This last scenario is given by Eq 6:

$$Reduction(y_{k})\;=\;{\left(1\;-\;Reduction(y_{10})\right)}^{\frac{10-k}{k}}\times Reduction(y_{10})$$ (6)

See Supporting Information S1 Methods section 2.3 for more details.

## Results

Table 1 shows baseline characteristics of the Mexican adults. The final sample included 3,005 individuals, which expanded to 64,885,715 adults. The average added sugar consumption from all sources was 244.9 kcal/day (12.6%) and from SSBs was 101.1 kcal/day (5.1% TEI). Men consumed more added sugar in total and from SSBs. Young adults, 20 to 39 years old, were the largest consumers of added sugar in total and from SSBs in comparison with other age groups. Smaller differences in added sugar consumption were observed across SES groups; in general, the low SES group had the lowest consumption of total and SSB added sugars.

**Table 1:** Sample characteristics, added-sugar intake (total and from SSBs) in and their contribution to the %TEI for the adult population in Mexico before regulation.

	Proportion of individuals in the sample (%)	Total added-sugar intake (kcal/day) (95% CI)	%TEI	Added-sugar intake from SSBs (kcal/day) (95% CI)	%TEI
**Overall**		244.9 (232.9–256.9)	12.6	101.1 (93.3–109.0)	5.1
**Sex**					
Male	47.7	278.9 (259.9–297.9)	12.9	128.0 (115.4–140.6)	6.1
Female	52.3	213.9 (199.6–228.2)	12.2	76.7 (67.4–86.0)	4.2
**Age group**					
20–39	44.6	285.7 (264.6–306.8)	13.8	130.8 (117.5–144.2)	6.4
40–59	36.8	235.7 (217.4–254.1)	12.2	90.3 (78.1–102.4)	4.5
60+	18.6	165.2 (148.2–182.2)	10.3	51.4 (39.3–63.5)	3.2
**SES**					
Low	29.7	213.1 (196.0–230.2)	11.3	84.5 (71.8–97.2)	4.5
Middle	30.1	255.0 (233.1–277.0)	12.7	118.0 (102.6–133.3)	5.7
High	40.2	260.7 (239.3–282.2)	13.4	100.8 (87.6–114.0)	5.2

**Abbreviations:** %TEI, percent of total energy intake; SES, socioeconomic status; SSB, sugar-sweetened beverage.

Table 2 shows the estimated results of halving the sugar content in SSBs. Twelve years after implementation, assuming 43% compensation, the energy intake could be reduced by 28.8 kcal/day (95% CI 26.6–31.1), which translates into a 1.3 kg (95% CI 1.2–1.4) reduction in weight and a 0.5 kg/m^2^ (95% CI 0.5–0.6) decrease in BMI. Stratifying by sex, the sugar regulation is estimated to have larger weight decreases in males (1.6 kg; 95% CI 1.4–1.7) than in females (1.1 kg; 95% CI 0.9–1.2). Younger adults, 20 to 39 years old, experience larger decreases in weight (1.6 kg; 95% CI 1.5–1.8) compared to older adults (0.7 kg; 95% CI 0.5–0.9). Stratifying by SES, people in the middle SES group are expected to experience larger weight decreases (1.5 kg; 95% CI 1.3–1.7) compared to low SES (1.1 kg; 95% CI 0.9–1.2) and high SES (1.3 kg; 95% CI 1.2–1.5).

**Table 2:** Predicted reduction in added sugars, energy intake, body weight, and BMI from a 50% gradual reduction of added sugars to SSBs, considering 43% compensation after 12 years.

	Added sugars (kcal/day) (95% CI)	Energy intake* (kcal/day) (95% CI)	Weight (kg) (95% CI)	BMI (kg/m^2^) (95% CI)
**Average**	−50.6 (−54.5 to −46.6)	−28.8 (−31.1 to −26.6)	−1.3 (−1.4 to −1.2)	−0.5 (−0.6 to −0.5)
**Sex**				
Male	−64.0 (−70.3 to −57.7)	−36.5 (−40.1 to −32.9)	−1.6 (−1.7 to −1.4)	−0.6 (−0.6 to −0.5)
Female	−38.3 (−43.0 to −33.7)	−21.8 (−24.5 to −19.2)	−1.1 (−1.2 to −0.9)	−0.5 (−0.5 to −0.4)
**Age group**				
20–39	−65.4 (−72.1 to −58.7)	−37.3 (−41.1 to −33.5)	−1.6 (−1.8 to −1.5)	−0.6 (−0.7 to −0.6)
40–59	−45.1 (−51.2 to −39.1)	−25.7 (−29.2 to −22.3)	−1.2 (−1.4 to −1.1)	−0.5 (−0.6 to −0.4)
60+	−25.7 (−31.8 to −19.7)	−14.7 (−18.1 to −11.2)	−0.7 (−0.9 to −0.5)	−0.3 (−0.3 to −0.2)
**SES**				
Low	−42.2 (−48.6 to −35.9)	−24.1 (−27.7 to −20.5)	−1.1 (−1.2 to −0.9)	−0.4 (−0.5 to −0.4)
Middle	−59.0 (−66.7 to −51.3)	−33.6 (−38.0 to −29.2)	−1.5 (−1.7 to −1.3)	−0.6 (−0.7 to −0.5)
High	−50.4 (−57.0 to −43.8)	−28.7 (−32.5 to −25.0)	−1.3 (−1.5 to −1.2)	−0.5 (−0.6 to −0.4)

*Net energetic change assuming 43% compensation for sources other than added sugars.

**Abbreviations:** BMI, body mass index; SES, socioeconomic status; SSB, sugar-sweetened beverage.

Table 3 shows the expected change in the prevalence and prevented cases of overweight and obesity 12 years after the implementation of the sugar regulation. The regulation could increase the prevalence of people with normal BMI by 3.8 pp (95% CI 2.8–4.9), which translates into 3.5 million individuals more with normal weight by the year 2032, along with a reduction in obesity of 3.9 pp (95% CI 2.6–5.1), which translates into 3.5 million less individuals in the obesity category by 2032. Larger reductions in obesity are expected for adults 20 to 39 years old (4.7 pp; 95% CI 2.8–6.6) than for those 40 to 59 years old (4.1 pp; 95% CI 2.0–6.3) or older adults (1.3 pp; 95% CI 0.4–2.3). People in the low SES group are expected to experience the highest decrease in obesity (4.8 pp; 95% CI 2.1–7.6) followed by the high SES (3.7 pp; 95% CI 2.0–5.5) and middle SES (3.1 pp; 95% CI 1.4–4.8) groups. However, the middle SES group could also experience the largest increases in the prevalence of normal weight (4.3 pp; 95% CI 2.2–6.5) followed by high SES (3.8 pp; 95% CI 2.3–5.4) and low SES (3.4 pp; 95% CI 1.8–5.0).

**Table 3:** Predicted change in absolute and relative prevalence and absolute number of individuals for normal, overweight, and obesity after 12 years of the implementation of a 50% gradual reduction of added sugar in SSBs, considering a 43% compensation (data projected to year 2032).

		Baseline prevalence (%; 95% CI)	Change in prevalence (absolute pp; 95% CI)	Percent change in prevalence (% baseline; 95% CI)	Change in number of individuals (in millions; 95% CI)
**Average**	Normal	30.4 (27.9 to 33.1)	3.8 (2.8 to 4.9)	12.6 (9.0 to 16.3)	3.5 (2.6 to 4.5)
	Overweight	38.7 (36.1 to 41.4)	0.0 (−1.6 to 1.7)	0.0 (−4.2 to 4.3)	0.0 (−1.5 to 1.5)
	Obesity	30.9 (28.5 to 33.3)	−3.9 (−5.1 to −2.6)	−12.5 (−16.2 to −8.8)	−3.5 (−4.7 to −2.4)
**Sex**					
Male	Normal	36.4 (32.3 to 40.7)	5.0 (3.5 to 6.5)	13.7 (9.0 to 18.3)	2.2 (1.5 to 2.8)
	Overweight	39.5 (35.4 to 43.7)	−1.0 (−3.5 to 1.4)	−2.6 (−8.7 to 3.6)	−0.4 (−1.5 to 0.6)
	Obesity	24.1 (20.9 to 27.7)	−4.0 (−5.8 to −2.1)	−16.4 (−23.4 to −9.4)	−1.7 (−2.5 to −0.9)
Female	Normal	25.0 (22.2 to 28.0)	2.8 (1.5 to 4.2)	11.3 (5.6 to 17.0)	1.4 (0.7 to 2.0)
	Overweight	38.0 (34.4 to 41.7)	1.0 (−1.2 to 3.1)	2.5 (−3.3 to 8.3)	0.5 (−0.6 to 1.5)
	Obesity	37.0 (33.7 to 40.4)	−3.8 (−5.4 to −2.2)	−10.2 (−14.3 to −6.1)	−1.8 (−2.6 to −1.1)
**Age group**					
20–39	Normal	39.4 (35.4 to 43.6)	4.9 (3.0 to 6.9)	12.5 (7.2 to 17.9)	2.0 (1.2 to 2.8)
	Overweight	35.2 (31.2 to 39.5)	−0.3 (−3.1 to 2.6)	−0.7 (−8.7 to 7.3)	−0.1 (−1.3 to 1.1)
	Obesity	25.3 (22.2 to 28.8)	−4.7 (−6.6 to −2.8)	−18.5 (−25.5 to −11.5)	−1.9 (−2.7 to −1.1)
40–59	Normal	19.1 (15.9 to 22.9)	3.6 (2.1 to 5.1)	18.8 (9.9 to 27.6)	1.2 (0.7 to 1.7)
	Overweight	41.2 (36.8 to 45.7)	0.5 (−2.2 to 3.3)	1.3 (−5.3 to 7.9)	0.2 (−0.7 to 1.1)
	Obesity	39.7 (35.4 to 44.1)	−4.1 (−6.3 to −2.0)	−10.4 (−15.7 to −5.2)	−1.4 (−2.1 to −0.7)
60+	Normal	31.3 (26.5 to 36.4)	1.7 (0.7 to 2.7)	5.5 (2.0 to 8.9)	0.3 (0.1 to 0.5)
	Overweight	42.1 (37.0 to 47.4)	−0.4 (−1.8 to 1.0)	−0.9 (−4.3 to 2.5)	−0.1 (−0.3 to 0.2)
	Obesity	26.6 (22.5 to 31.3)	−1.3 (−2.3 to −0.4)	−5.0 (−8.6 to −1.3)	−0.2 (−0.4 to −0.1)
**SES**					
Low	Normal	37.9 (33.4 to 42.6)	3.4 (1.8 to 5.0)	8.9 (4.4 to 13.4)	0.9 (0.5 to 1.3)
	Overweight	35.3 (31.1 to 39.7)	1.4 (−1.8 to 4.7)	4.1 (−5.3 to 13.4)	0.4 (−0.5 to 1.3)
	Obesity	26.8 (22.9 to 31.1)	−4.8 (−7.6 to −2.1)	−18.0 (−26.9 to −9.0)	−1.3 (−2.1 to −0.6)
Middle	Normal	30.6 (26.4 to 35.1)	4.3 (2.2 to 6.5)	14.2 (6.4 to 22.0)	1.2 (0.6 to 1.8)
	Overweight	37.1 (33.0 to 41.4)	−1.2 (−4.1 to 1.6)	−3.3 (−10.9 to 4.2)	−0.3 (−1.1 to 0.4)
	Obesity	32.3 (28.4 to 36.5)	−3.1 (−4.8 to −1.4)	−9.6 (−14.7 to −4.5)	−0.9 (−1.3 to −0.4)
High	Normal	24.8 (20.6 to 29.6)	3.8 (2.3 to 5.4)	15.4 (8.2 to 22.5)	1.4 (0.8 to 2.0)
	Overweight	42.4 (37.7 to 47.3)	−0.1 (−2.5 to 2.3)	−0.2 (−5.9 to 5.5)	0.0 (−0.9 to 0.9)
	Obesity	32.7 (28.5 to 37.2)	−3.7 (−5.5 to −2.0)	−11.4 (−16.5 to −6.3)	−1.4 (−2.0 to −0.7)

pp to reflect the absolute difference.

“Normal” includes underweight <18.5 kg/m^2^.

**Abbreviations:** pp, percentage points; SES, socioeconomic status; SSB, sugar-sweetened beverage.

**Fig 1:**
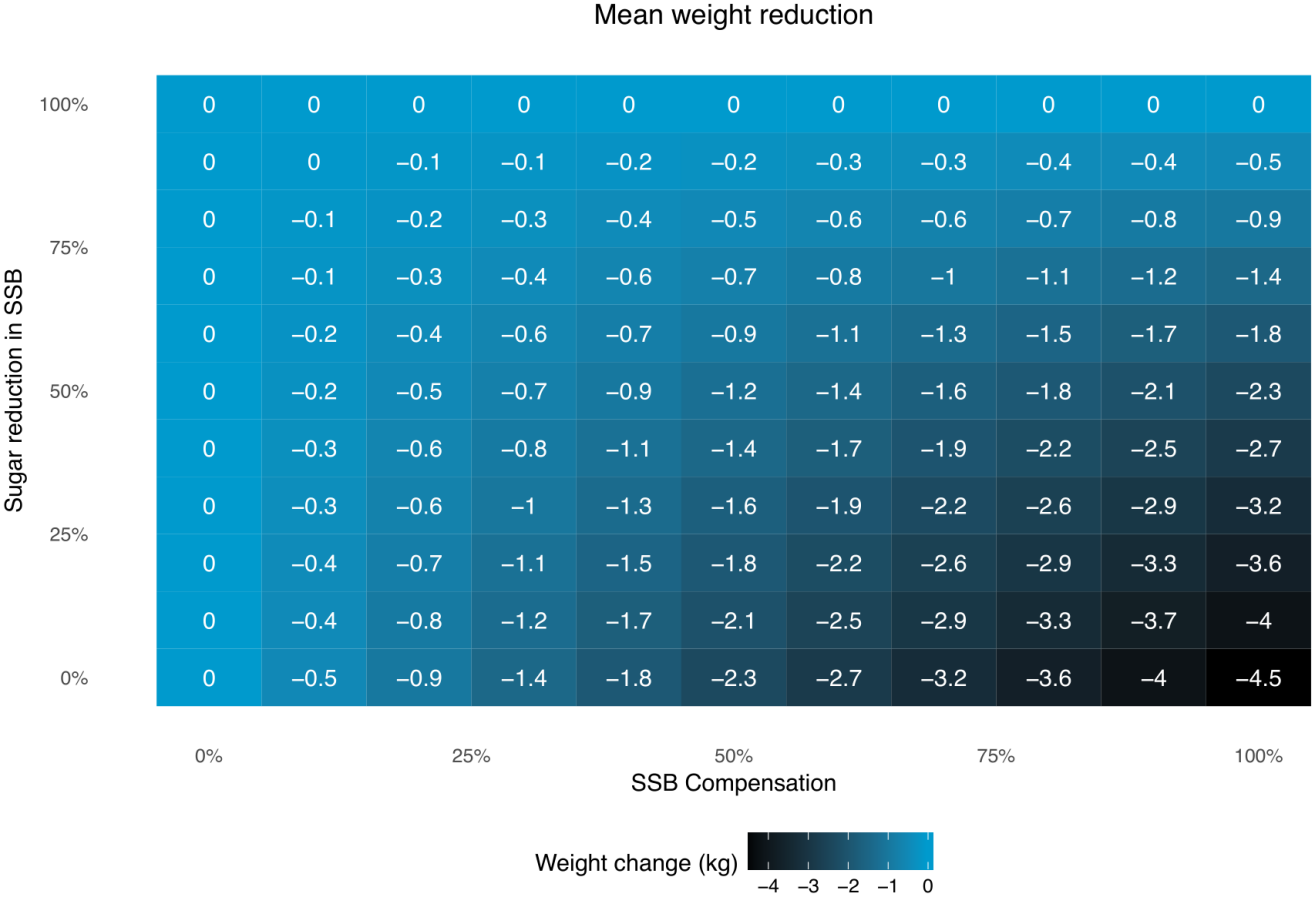
Sensitivity analysis for estimated weight change after 12 years proposing different sugar reductions and different compensation rates. SSB, sugar-sweetened beverage.

Fig 1 shows a sensitivity analysis measuring the impact of several sugar reduction targets, in combination with different compensation rates. Our main analysis considers a 50% reduction in SSBs with 43% compensation, which results in a 1.3 kg reduction after 12 years; this effect could be 0 kg with 100% compensation or an increase of up to 2.3 kg assuming no compensation. Considering no compensation, or compensation with noncaloric beverages, reducing sugar content in SSBs from 0% to 100% could reduce body weight by between 0 and 4.5 kg, respectively.

## Discussion

We estimated the impact of reformulating SSBs to reduce added sugars and achieve an average 10% of the TEI in the Mexican adult population. This goal would be achieved if added sugars in SSBs were reduced by 50%. Assuming that halving the sugar content takes up to 10 years and a 43% caloric compensation, this regulation would produce a reduction of 1.3 kg of body weight and 0.5 kg/m^2^ of BMI, reducing by 3.9 pp the prevalence of obesity (12.6% relative to the baseline). Larger decreases will be expected among males, young adults, and the middle SES group.

Regulating added sugars through national reformulation has been described as cost-effective, in some cases having a larger impact than reducing portion sizes [17,39,40]. Several countries have forecasted the long-term impact of reformulating SSBs to reduce sugar content. In Australia, a mandatory reduction of added sugar by 30% in SSBs (assuming no compensation) was estimated to produce a 1.39 kg reduction in weight (0.56 kg/m^2^ of BMI) over a lifetime [39]. In the UK, Ma and colleagues estimated that reducing 40% of added sugar in SSBs assuming no compensation would decrease body weight (−1.2 kg), overweight (−1 pp), and obesity (−2.1 pp) in adults after 5 years [41]. Another study in the UK by Briggs and colleagues forecasted a 0.9% reduction in obesity prevalence (−0.2 pp) by reducing 30% added sugar on high-sugar drinks and 15% in medium-sugar drinks [17]. This study included the overall UK population and used comparative risk assessment to estimate weight change using parameters from meta-analytical estimates obtained by combining two randomized controlled trials (RCTs) that lasted 6 months. While RCTs are the gold standard, fitting linear models might not be suitable for long-term effect evaluation. Our results are in line with those of Australia and the study by Ma and colleagues in the UK [39,41]. Assuming no compensation, gradually halving added sugars in SSBs in Mexico could reduce body weight by 2.3 kg (−0.9 kg/m^2^) and the obesity prevalence by 6.3 pp after 12 years (Table A in S1 Results). We estimated the impact of the SSB reformulation in people who reported drinking SSBs in the past 24 hours (51.8% of the adult population), who are more likely to be affected by the intervention (Table B in S1 Results); among them, added sugar reformulation could produce a weight reduction of 2.5 kg (−7.5 pp in obesity, −22.9% with respect to baseline). While the 24-hour recall underestimates the prevalence of SSB consumers, these estimates show that larger benefits of the reformulation are to be expected for people who frequently consume SSBs. These results reflect the importance of regulating added sugars in SSBs to decrease body weight and the prevalence of overweight and obesity.

Body weight modeling is a complex task, as many components and interrelationships need to be considered. In this paper, we used the model proposed by Chow and Hall, which has been widely used to estimate the impact of nutritional policies, including the potential impact of sugar reformulation in SSBs [41]. Chow and Hall’s model is a physiological model that has been validated against experimental data and can be implemented using individual-level or aggregated data; however, other models to estimate weight change are available. Christiansen and Garby proposed an individual-based model that expresses body weight as a function of energy intake and physical activity level and can also be implemented at an individual level [42]. Briggs and colleagues used experimental data from RCTs to estimate the potential impact of sugar reformulation in the UK using the comparative assessment framework [17]. To reflect the heterogeneity of models, we conducted a sensitivity analysis following Briggs methodology, a modified version of Briggs using effect estimates of SSB consumption on body weight in Mexican women [43] and Christiansen and Garby’s model [42]. The estimates obtained using comparative risk assessment are smaller than our results (see Table C in S1 Results): under Briggs approach, the expected body weight reduction would reach 0.12 kg after 6 months, increasing to 0.44 kg at 2 years if the association of SSB consumption and weight in Mexican women is used as the effect estimate; in contrast, Christiansen and Garby’s model with 43% compensation produces a higher estimate than ours (1.52 kg compared to 1.31 kg). The comparison across models is difficult because they consider different time frames; still, we could consider Briggs to be the lowest boundary and Christiansen the highest.

### Implementation process

Sugar reductions to SSBs have been previously proposed, although the mechanism for implementation and enforcement has not been clearly laid out other than the UK study on reformulation by use of a tiered sugar tax [17]. Sugar reduction could be part of a self-regulatory plan or a national regulation. Industry self-regulation has proven to be ineffective because its main focus is usually restricted to improve public perception: an image of concern and care is created, while at the same time, standards and enforcement are relaxed [44]. In contrast, a regulation enforced by the government has proven to be effective to limit food additives. For instance, trans fat bans in New York restaurants led to a reduction in trans fat consumption [45]. Also, government programs to reduce salt content in industrialized products produced significant decreases in salt consumption in China, Finland, France, Ireland, South Africa, and the UK [46,47]. The UK, Ireland, and South Africa are three countries that are using tiered and/or tiered plus grams of sugar tax structures to promote reformulation of SSBs [17,48]. Consequently, we consider that a national regulation to halve the sugar content over 10 years is feasible and necessary to reduce obesity in Mexico and should be promoted along with other simultaneous interventions, such as food taxes, information campaigns, improvement of food systems, and strengthening of product labeling [13,49]. In our opinion, sugar reduction in SSBs should be implemented as a national regulation to avoid pressure from industry, provide compliance, provide monitoring, and establish independent evaluations of effectiveness.

To be successful a state-enforced sugar reduction requires a careful analysis of the implementation process, taking into the account the consumer’s preference and the impact on flavor. Recent evidence suggests that small reductions in added sugar content in SSBs have no effect on acceptance or pleasantness and could further increase the preference for less sweetened food or beverages [50,51]. Furthermore, experimental evidence has shown low ability of consumers to discriminate across SSB brands or type of sweetener [52,53]. In our models, the decreasing reduction scenario, which sets larger reductions on sugar content at the beginning, had the highest impact on weight at year 10; however, this scenario could imply a stronger change in flavor. The other two scenarios (Fig A in S1 Results) imply smaller sugar reductions at the beginning and could be more easily accepted by consumers, achieving the same weight reductions than the decreasing reduction scenario by year 12. Further research is needed to estimate the effect of lowering added sugars on product preference, acceptance, and consumption to inform the best implementation scenario and improve the odds of success.

Finally, a state-enforced sugar regulation must carefully weigh the use of noncaloric sweeteners (NNS). Advocates of NNSs propose their use to maintain the same level of sweetness while decreasing caloric content. In this study, we assumed no substitution by NNS given reported unpleasant taste or a slower-onset sweetness in comparison with sugar [51]. Also, in recent complex research, cohort studies suggest that NNS could increase caloric intake and body weight, as well as hypertension, metabolic syndrome, type 2 diabetes, and cardiovascular events [54,55]. Studies that control for diet modification and RCTs suggest no effect, leading dietary guideline committees in the US and UK and other organizations to continue to recommend the intake of NNSs [11,55–58]. Other concerns relate to NNS addictiveness as a result of an increase in sugar tolerance and the overstimulation of the sugar receptors, yet RCT results suggest no effect [59–62]. NNS addictiveness results are often taken from mice models, which might be an inappropriate comparison because NNS concentration by mice per body weight have about 100 times the sweetness preference of humans. Although the negative evidence of NNS is still insufficient, ideally, we would want the sweetness threshold to be reduced and—where potable water is available—have people shift to less sweetened beverages and nonsweetened beverages.

### Strengths and limitations

Some limitations for input data must be acknowledged. First, both BMI and sugar intake from SSBs come from nationally representative data. We used a single 24-hour dietary recall to estimate sugar intake at the population level and subgroups; the 24-hour recall is limited to representing usual individual intake as it fails to capture day-to-day variation, yet it is adequate to estimate energy means at an aggregated level [63]. Also, the 24-hour recall could underestimate sugar intake (a source of variability that is not included in the model), particularly in individuals with higher BMI; however, if anything, this error would lead to conservative estimates of the regulation effect [64]. ENSANUT 2012 obtained dietary recall from all seasons, except for summer (June to September), which ignores the variations in consumption observed in those months. In summer, SSB consumption is higher, which could lead to the underestimation of the total effect of the sugar regulation [65]. Finally, we excluded 164 observations from the ENSANUT sample due to missing anthropometric data. Excluded observations were older and more men but had comparable total and SSB-related caloric intakes to the analytical sample; given the small proportion of missing observations and the similar caloric intake, we assume that these exclusions will not fundamentally change our estimates.

Some limitations and strengths for the modeling process must be acknowledged. In our main scenario, we assume steady-state conditions along the 12-year simulation and a 43% compensation, but under the assumption that the industry would not interfere with the caloric reduction derived from sugar reductions. However, the industry could find ways to circumvent the regulation, such as increasing the black market, in which case our primary scenario would overestimate the expected weight decreases. A possible downside of the SSB regulation would be the increase of the prevalence of underweight (BMI < 18.5 kg/m^2^). We included underweight in the normal BMI group due to its small percentage and to facilitate the presentation of results; in Mexico, a very small percentage of individuals are underweight (0.8%), but following reformulation, it could increase to 1.7%. Low body weight in adults can be produced by undernutrition but also by medical conditions such as cancer, diabetes, or anorexia; the inclusion of these conditions is beyond the scope of our paper, although the potential increase in underweight following reformulation should be considered to provide adequate detection and medical care to underweight individuals. Modeling the impact of reformulation in children and adolescents is important, given that SSBs contribute to a large proportion of their energy consumption [10]. However, weight modeling in these age groups is challenging because it requires additional energy functions to take into account growth [66]; unfortunately, no such functions are available for Mexican children and adolescents. Finally, Hall’s model does not provide CIs to the estimated weight change, therefore our CIs only consider the sources of error captured by the survey data. However, this model has been previously validated and used to estimate the impact of SSB tax in Mexico [67]; also, it has the advantage of using individual-level data, which allows for result stratification.

## Conclusions

Increasing awareness about excessive sugar contents and implementing effective policies to decrease its consumption is paramount to prevent obesity. Considering the variables involved, providing a precise prediction of the impact of sugar reformulation in beverages is difficult; our study suggests that reformulation could reduce obesity by 12.5%, yet this result could vary depending on implementation conditions, caloric substitution, and industry response. National regulation of sugar content is an effective and feasible action that could be implemented in Mexico and other countries, to gradually adapt the population taste to lower sugar intake and promote healthier diets.

## Supporting information

S1 Methods Supporting information for the input data, estimation of the sugar reduction target, implementation of the weight change model, and sensitivity analysis. (PDF)

S1 Results Supporting information for the impact of sugar reduction program using different assumptions. (DOCX)

## Abbreviations

BMI: body mass index
CONAPO: Mexico National Population Council
ENSANUT: Mexico National Health and Nutrition Survey
NNS: noncaloric sweetener
pp: percentage points
RCT: randomized controlled trial
SES: socioeconomic status
SSB: sugar-sweetened beverage
TEI: total energy intake
