# Correction: Expected changes in obesity after reformulation to reduce added sugars in beverages: A modeling study

**DOI:** 10.1371/journal.pmed.1002743

**Published:** 2019-01-24

**Authors:** Ana Basto-Abreu, Ariela Braverman-Bronstein, Dalia Camacho-García-Formentí, Rodrigo Zepeda-Tello, Barry M. Popkin, Juan Rivera-Dommarco, Mauricio Hernández-Ávila, Tonatiuh Barrientos-Gutiérrez

There is an error in the reference for the source of the mathematical model used in this manuscript. The authors cited Chow&Hall 2008 instead of Hall et al. 2011. Both articles have Hall in their authorship. While Chow&Hall (2008) explains the model conceptualization and the model behavior with non-real simulations, Hall et al. (2011) explains the model implementation to the adult population with real data. The correct citation for reference #27 should read:

27. Hall KD, Sacks G, Chandramohan D, Chow CC, Wang YC, Gortmaker SL, et al. Quantification of the effect of energy imbalance on bodyweight. Lancet. 2011;378: 826–837. doi:10.1016/S0140-6736(11)60812-X

The reference is made several times throughout the paper, and therefore the following should be corrected:

The Data Availability Statement should read: Baseline added sugar consumption from SSBs and weight were obtained from the 2012 Mexico National Health and Nutrition Survey (http://ensanut.insp.mx/). The final dataset after running the Hall and colleagues’ model is available at the open science framework, available at https://osf.io/vfcm8/ (DOI: 10.17605/OSF.IO/VFCM8). The mathematical model used to estimate the impact on body weight is freely available within an R package version 1.0.0 named ‘bw’ in https://cran.r-project.org/web/packages/bw/index.html.

The first sentence in the subsection of the Methods section entitled “Change in body weight and obesity prevalence” should read: “The change in body weight was estimated using the dynamic weight change model proposed by Hall and colleagues [27], implemented individual by individual for all participants in the ENSANUT. This model accounts for the dynamic physiological adaptation that occurs following weight loss, and it has been validated with experimental data [27–29].” The first sentence of the second paragraph in that same section should read: “Using the expected reduction in energy intake, we implemented Hall and colleagues’ model to each adult in ENSANUT 2012 to obtain the expected reduction in weight after 12 years (considering a 2-year time lag for caloric changes to influence weight).”

The first three sentences of the third paragraph in the Discussion section should read: Body weight modeling is a complex task, as many components and interrelationships need to be considered. In this paper, we used the model proposed by Hall and colleagues, which has been widely used to estimate the impact of nutritional policies, including the potential impact of sugar reformulation in SSBs [41]. Hall and colleagues’ model is a physiological model that has been validated against experimental data and can be implemented using individual-level or aggregated data; however, other models to estimate weight change are available.”
