# Supplementary material for: Expected changes in obesity after reformulation to reduce added sugars in beverages: A modeling study
Source: PLoS Med. 2018 Oct 5;15(10):e1002664. doi: 10.1371/journal.pmed.1002664 (PMC6173390; doi:10.1371/journal.pmed.1002664)
Supplement: S1 Methods — (PDF) [file pmed.1002664.s001.pdf]

Supplementary methods (S1 methods) to “Regulating added sugars in sweetened beverages could curb the obesity epidemic in Mexico: a modeling study”

Contents

1 Data collection **5**
1.1 Included beverages in the regulation . . . 5
1.2 Survey design . . . 5

2 Estimation of the sugar reduction target for regulation **6**
2.1 Formula derivation . . . 6
2.2 Individual estimation to obtain added-sugar percent reduction . . . 6
2.3 Regulation scenarios . . . 7
2.3.1 Decreasing scenario . . . 7
2.3.2 Increasing scenario . . . 8
2.3.3 Constant scenario . . . 8
2.4 Individual modelling of energy intake for reduction scenarios . . . 8

3 Weight change model **9**
3.1 Individual implementation . . . 10

4 Sensitivity analysis **12**
4.1 Different scenarios . . . 12
4.2 Model under compensation assumptions . . . 12

Notation

- **SSBs**, sugar sweetened beverages.
- **US**, United States.
- **UK**, United Kingdom.
- **TEI**, total energy intake. See TEI_{init} for total energy intake at baseline.
- **ENSANUT**, National Health and Nutrition Survey.
- **INSP**, National Institute of Public Health.
- **BMI**, body mass index.
- **WHO**, World Health Organization.
- TEI_{init}, total energy intake at baseline.
- SSB_{max}, maximum consumption of kcals from SSB such that 10% added sugar is achieved.
- κ_p, percent added sugar desired.
- Others, consumption of other added sugars (kcal).
- SSB_{init}, current added sugar consumption from SSB (kcal).
- Δc, theoretical consumption change to achieve $\kappa_p \times 100\%$ consumption of added sugar.
- i **(superscript)**, ith individual in the sample. $TEI_{\text{init}}^{(i)}$ corresponds to total energy intake at baseline for individual i.
- $\overline{\text{prop}}$, estimated average proportional change for SSBs consumers such that $\kappa_p \times 100\%$ added sugar is achieved.
- $y_1, y_2, \ldots, y_{10}$, year 1, year 2, year 10 (respectively).
- Reduction(y_k), reduction of sugar % at year k.
- λ, proportion of added sugar reduction that one desires to achieve by year ℓ.
- ℓ, year at which a $\lambda \times 100\%$ added sugar reduction is achieved.
- t, variable for day.
- BW, body weight function (kg) as a function of time ($BW \equiv BW(t)$).
- ECF, extracellular fluid (kg) as a function of time ($ECF \equiv ECF(t)$)
- G, glycogen reservoire (kg) as a function of time ($G \equiv G(t)$).
- F, fat mass (kg) as function of time ($F \equiv F(t)$).
- L, lean mass (kg) as function of time ($L \equiv L(t)$).
- EE, energy expenditure as function of time ($EE \equiv EE(t)$).
- AT, adaptative thermogenesis as a function of time ($AT \equiv AT(t)$).
- RMR_{init}, resting metabolic rate at baseline.
- F_{init}, fat mass at baseline.
- H_{init}, height at baseline.

- ΔTEI, change in energy intake as a function of time ($\Delta TEI \equiv \Delta TEI(t)$).
- K, constant for the initial energy balance condition (equilibrium).
- p, proportion of lean mass attributable to energy intake/expenditure difference.
- PAL, physical activity level.
- TEF, thermal effect of feeding.

1 Data collection

We used data from the National Health and Nutrition Survey (ENSANUT) from 2012 which is available in [1]. The final dataset called "Database Sugar regulation Mexico.dta" can be found at the Open Science Framework [2] with the DOI: 10.17605/OSF.IO/VFCM8 (web link osf.io/vfcm8). Table A details the variables in the dataset.

Name	Variable
`intp`	Identifier for each individual in ENSANUT's dataset.
`agecat`	Age category. Levels: "20 to 40", "40 to 60", "Over 60".
`code_upm`	Identifier of primary sampling unit.
`pondef_a`	Complex survey weight.
`est_var`	Strata for the estimation of variances accounting for survey design.
`folio`	Unique identifier for each household in ENSANUT's dataset (to merge with anthropometric databases).
`folio_c2`	Unique identifier for each household in ENSANUT's dataset (to merge with nutritional databases).
`sex`	Sex of the individual, coded as 1: "male"; 2: "female".
`age`	Age (yrs).
`weight_baseline`	Weight at baseline (kg).
`height`	Height (cm).
`bmicategories`	Categories of "normal" (1), "overweight" (2) and "obesity" (3) at baseline.
`ses`	Socioeconomic level, divided in tertiles, using the weighted sample.
`sugar_ssb`	Added sugar consumption at baseline from SSB (kcal).
`kcaltot`	Daily total caloric intake at baseline (kcal).
`finalweight`	Weight after intervention (kg).
`changekcal`	Change in energy after intervention (kcal).
`changeweight`	Change in weight after 12 years (kg).
`bmifinal`	Body mass index after 12 years (kg/m^2).
`changebmi`	Change in body mass index after 12 years (kg/m^2).
`final_bmiprevalences`	Categories of "normal" (1), "overweight" (2) and "obesity" (3) after intervention.
`sugar_tot`	Added sugar consumption from all sources before intervention (kcal).

Table A: *Variables in `Database_Sugar regulation Mexico.dta`*

1.1 Included beverages in the regulation

Table B shows the beverages included not included in the sugar regulation. The beverages classification is the same as in Sanchéz-Pimienta, et al which states: [3]

> "Regular soda" includes all brands of carbonated sodas with caloric sweeteners; "Fruit, flavored, sports, and energy drinks" include noncarbonated flavored water, industrialized juice, and energy and sport drinks; "sweetened coffee and tea" include coffee and tea with caloric sweeteners; "aguas frescas and homemade SSBs" include aguas frescas frescas, a traditional flavored water-based preparation, and fruit shakes without sugar or other caloric sweeteners, atoles without milk, and pozol (fermented corn beverage); and "sweetened milk and milk beverages" include milk, milk shakes, smoothies, coffee or tea made with milk (more than one-third of the preparation), and atoles with milk.[3]

1.2 Survey design

ENSANUT is a cross-sectional, multi-stage, probabilistic survey representative of the Mexican population survey whose methodology has been explained elsewhere [4]. To account for this design, we used the `R` [5] package `survey` [6] with the following design:

```
svystr <- svydesign(id = ~id , strata = ~est_var, weights = ~pondef_a, PSU = ~code_upm,
                    data = Adults)

options(survey.lonely.psu = "adjust")
```

The same design in Stata [7], is achieved by:

```
svyset [w = pondef_a], psu(code_upm) strata(est_var) singleunit(centered)
```

All population-level estimations were done considering this design in either of the programmes.

Sugar Regulation	Beverages group	Description
**Included**	*Industrialized carbonated beverages*	Regular soda.
	Industrialized non-carbonated beverages	Fruit, flavored, sports, and energy drinks.
**Not Included**	*Homemade sweetened beverages*	Sweetened coffee and tea, aguas frescas and homemade SSBs.
	Dairy beverages	Sweetened milk and milk beverages

Table B: *Beverages included in the regulation.*

2 Estimation of the sugar reduction target for regulation

2.1 Formula derivation

To estimate the target for added sugar regulation in SSBs, we estimated the maximum added sugar intake from SSBs such that overall consumption of added sugar was under the WHO guidelines. These guidelines establish that at most, 10% of the total energy intake (TEI_{init}) should come from added sugars. To find the target, we considered only those individuals that reported a consumption > 0 and we calculated the amount of added sugar from SSBs and from other sources as well as the Total Energy Intake in kcals ($TTEI_{\text{init}}$). To estimate the individual level of maximum consumption of kcals from SSBs (SSB_{max}), such that a 10% added sugar is achieved, we set $\kappa_p = 0.1$ (10% of total energy intake coming from added sugar). This was specified in the following equation:

$$\kappa_p = \frac{\text{SSB}_{\text{max}} + \text{Others}}{TEI_{\text{init}} + (\text{SSB}_{\text{max}} - \text{SSB}_{\text{init}})} = 0.1, \tag{1}$$

where Others is the consumption of other added sugars (kcal), TEI_{init} is the current total energy intake (kcal), and SSB_{init} is the current added sugar consumption from SSBs (kcal). The maximum sugar consumption from SSBs (SSB_{max}) hence equals:

$$\text{SSB}_{\text{max}} = \frac{\kappa_p}{1 - \kappa_p} \cdot (TEI_{\text{init}} - \text{SSB}_{\text{init}}) - \frac{1}{(1 - \kappa_p)} \cdot \text{Others}. \tag{2}$$

Using the SSB_{max}, we obtained the theoretical consumption change, Δ_{C}:

$$\Delta_{\text{C}} = \begin{cases} 0, & \text{if } 0 \leq \text{SSB}_{\text{init}} \leq \text{SSB}_{\text{max}}, \\ -\text{SSB}_{\text{init}}, & \text{if } \text{SSB}_{\text{max}} \leq 0, \\ \text{SSB}_{\text{max}} - \text{SSB}_{\text{init}}, & \text{if } 0 < \text{SSB}_{\text{max}} < \text{SSB}_{\text{init}}. \end{cases} \tag{3}$$

Intuitively, if current sugar consumption from SSBs (SSB_{init}) was lower than the maximum consumption from SSBs (SSB_{max}), we kept consumption at the current level ($\Delta_{\text{C}} = 0$). If sugar from additional sources was above the 10% threshold, we reduced all sugar from SSBs ($\Delta_{\text{C}} = -\text{SSB}_{\text{init}}$). Finally, if by reducing SSBs to the SSB_{max} achieves the goal of 10% added sugars in overall energy intake, we reduced sugar consumption from SSBs to $\Delta_{\text{C}} = \text{SSB}_{\text{max}} - \text{SSB}_{\text{init}}$.

2.2 Individual estimation to obtain added-sugar percent reduction

For each individual, i, in the sample, we estimated their maximum amount of sugar from SSBs ($\text{SSB}^{(i)}_{\text{max}}$), such that the amount of added sugar in their total energy intake,$TEI^{(i)}_{\text{init}}$, is, at most, 10% for each individual. For that purpose, we used equation (2) and considered as inputs the individual's current total energy intake

($TEI^{(i)}_{\text{init}}$), their SSBs caloric intake ($\text{SSB}^{(i)}_{\text{init}}$), and their caloric intake from other added sugars ($\text{Others}^{(i)}$). The latter stand for `kcaltot`, `sugar_ssb`, and `sugar_tot` variables in our database.

Using each individual's $\text{SSB}^{(i)}_{\text{init}}$, and $\text{SSB}^{(i)}_{\text{max}}$ we obtained their theoretical consumption change $\Delta^{(i)}_{\text{C}}$. The individual proportional change of sugar consumption from SSB was then calculated as $\text{prop}^{(i)} = -\Delta^{(i)}_{\text{C}}/SSB^{(i)}_{\text{init}}$. We then estimated the average proportional change for consumers:[1] $\overline{\text{prop}} = 0.522$, which is equivalent to a reduction of 52.2% of sugar in SSB, which we rounded to 50%.

2.3 Regulation scenarios

After obtaining the λ% change of added sugar we then established different scenarios of regulation that would achieve said change. The decreasing scenario (section 2.3.1) was used for all analysis in the article (with $k = 50$) whilst the increasing (section 2.3.2) and constant (section 2.3.3) scenarios were used for the sensitivity analysis. As was shown in the sensitivity analysis; all scenarios converge after 12 years.

2.3.1 Decreasing scenario

Let $y_1, y_2, \ldots, y_{10}$ denote year 1, year 2 upto year 10 (respectively). The decreasing scenario assumes a yearly SSB-added-sugar reduction in which the yearly difference in added sugar% decreases in time. This scenario was implemented in [8] and is given by:

$$\text{Reduction}(y_1) = 1 - \big(1 - \text{Reduction}(y_k)\big)^{\frac{1}{k}}, \tag{4}$$

for $k = 1, 2, \ldots, 10$. The previous equation is equivalent to:

$$\text{Reduction}(y_k) = 1 - \big(1 - \text{Reduction}(y_1)\big)^{k}. \tag{5}$$

To achieve a $\lambda \times 100\%$ reduction by year y_ℓ one would plug in the λ in $\text{Reduction}(y_\ell)$ (4) to obtain the first year reduction associated to λ:

$$\text{Reduction}(y_1) = 1 - \big(1 - \lambda\big)^{\frac{1}{\ell}}, \tag{6}$$

and then substitute in (5) to obtain an expression for the reduction in year k:

$$\text{Reduction}(y_k) = 1 - \big(1 - \lambda\big)^{\frac{k}{\ell}}. \tag{7}$$

In the specific case of a a reduction of $\lambda = 0.5$ (50%) after 10 years, the k-th year formula is:

$$\text{Reduction}(y_k) = 1 - \big(0.5\big)^{\frac{k}{10}}. \tag{8}$$

The proportion reduced yearly from the original amount of sugar for a reduction of $\lambda = 0.5$ after 10 years is shown in Table C.

Year	y_1	y_2	y_3	y_4	y_5	y_6	y_7	y_8	y_9	y_{10}
Reduction	6.7%	12.9%	18.8%	24.2%	29.3%	34.0%	38.4%	42.6%	46.4%	50.0%
Difference	6.7%	6.2%	5.8%	5.4%	5.1%	4.7%	4.4%	4.1%	3.8%	3.6%

Table C: *Yearly proportion of sugar reduced from original amount of sugar for a reduction of $\lambda = 0.5$ after 10 years and difference in reduction % with previous year ($\text{Difference}(y_k) = \text{Reduction}(y_k) - \text{Reduction}(y_{k-1})$).*

[1] In accordance with the survey design established on section 1.2

2.3.2 Increasing scenario

This implementation scenario was done for sensitivity analysis. Instead of implementing a decrease in reduction of added sugar; we created a scenario in which the yearly difference in % reduction increases over time. In this case, the equation for the k-th year is given by:

$$\text{Reduction}(y_k) = \lambda \cdot \big(1 - \lambda\big)^{\frac{\ell - k}{k}}; \tag{9}$$

where $\lambda \times 100\%$ is the desired reduction by year ℓ. In the case of a 50% ($\lambda = 0.5$) SSB-added-sugar reduction by year 10 the specific equation for the k-th year is given by:

$$\text{Reduction}(y_k) = \frac{\big(0.5\big)^{\frac{10-k}{k}}}{2}, \tag{10}$$

which yields the values in Table D.

Year	y_1	y_2	y_3	y_4	y_5	y_6	y_7	y_8	y_9	y_{10}
Reduction	3.6%	7.4%	11.6%	16.0%	20.7%	25.8%	31.2%	37.1%	43.3%	50.0%
Difference	3.6%	3.8%	4.1%	4.4%	4.7%	5.1%	5.4%	5.8%	6.2%	6.7%

Table D: *Yearly proportion of sugar reduced using incremental scheme with a* 50% *reduction* ($\lambda = 0.5$) *after* $\ell = 10$ *years and difference in reduction % with previous year (Difference(*y_k*) = Reduction(*y_k*) − Reduction(*y_{k-1}*)).*

These reduction values are the ones implemented in the paper's sensitivity analysis (see section 4 below).

2.3.3 Constant scenario

This implementation scenario was done for sensitivity analysis (see section 4). In this scenario we implemented a constant % reduction such that if a $\lambda\%$ reduction is implemented over ℓ years then each year a $\lambda\%/\ell$ is reduced such that by year ℓ, $\lambda\%$ is achieved. For the specific case of $\lambda = 0.5$ (50% reduction) for $\ell = 10$ years the percent reduction is presented in Table E. The general formula for the k-th year in this case is given by:

Year	y_1	y_2	y_3	y_4	y_5	y_6	y_7	y_8	y_9	y_{10}
Reduction	5%	10%	15%	20%	25%	30%	35%	40%	45%	50.0%
Difference	5%	5%	5%	5%	5%	5%	5%	5%	5%	5%

Table E: *Yearly proportion of sugar reduced using the constant* $\frac{50}{10}\%$ *reduction and difference in reduction % with previous year (Difference(*y_k*) = Reduction(*y_k*) − Reduction(*y_{k-1}*)).*

$$\text{Reduction}(y_k) = k \cdot \frac{\lambda}{\ell}, \tag{11}$$

where λ denotes the expected reduction at year ℓ. In the particular case of a 50% reduction in 10 years (the one implemented in the paper's sensitivity analysis) the formula is:

$$\text{Reduction}(y_k) = k \cdot \frac{0.5}{10}. \tag{12}$$

2.4 Individual modelling of energy intake for reduction scenarios

The caloric reduction scenarios were designed globally. However, the reductions were conducted individually. Thus for each individual i we modelled their energy intake change at day t with a function $\Delta TEI^{(i)}$ given by:

$$\Delta TEI(t)^{(i)} = TEI^{(i)}_{\text{init}} - \text{Reduction}(y_{\lceil t/365 \rceil}) \cdot SSB^{(i)}_{\text{init}} \tag{13}$$

with $\lceil \cdot \rceil$ the ceiling function where $\lceil x \rceil$ stands for the smallest integer larger or equal to x. In the previous equation, $TEI^{(i)}_{\text{init}}$ stands for the total energy intake (kcals) of the i-th individual and $SSB^{(i)}_{\text{init}}$ their initial SSB consumption (kcals).

3 Weight change model

The weight change model [9] defines individual weight (kg) as the sum of fat F and lean mass L, extracellular fluid ECF and glycogen G:

$$BW = ECF + 3.7G + F + L. \tag{14}$$

Extracellular fluid $ECF \equiv ECF(t)$ is the solution to the ordinary differential equation system:

$$\begin{cases} \dfrac{dECF}{dt} & = \frac{1}{Na}\Big(\Delta Na_{diet} + \xi_{Na}(ECF - ECF_{init}) - \xi_{CI}(1 - CI/CI_b)\Big), \\ ECF_{init} & = 0. \end{cases} \tag{15}$$

where $Na = 3.22$ mg/ml, $\xi_{Na} = 3000$ mg/L/d, and $\xi_{CI} = 4000$ mg/d, are phisiological constants [9]. ΔNa_{diet} represents the change in sodium (mg/d) for the individual (3). CI_b is the carbohydrate intake at baseline (assumed to be 1/2 of energy intake estimated in (3)) and $CI \equiv CI(t)$ is the carbohydrate intake after the consumption reduction (assumed to be half the energy intake after reduction, $CI \equiv \Delta TEI(t)/2$). Glycogen mass $G \equiv G(t)$ is described by the ordinary differential equation:

$$\frac{dG}{dt} = \frac{1}{\rho_G}\Big(CI - k_G \cdot G^2\Big), \tag{16}$$

where $\rho_G = 4206.501$kcals/kg ($17.6 MJ/kg$), and $k_G = CI_b/G^2_{init}$ is a constant with $G_{init} = 0.5$ kg the initial glycogen mass.

Fat and lean mass, $F \equiv F(t)$ and $L \equiv L(t)$, represent the solutions to the following system of nonlinear ordinary differential equations:

$$\begin{aligned} \frac{dF}{dt} &= \frac{(1-p)}{\rho_F}\Big(TEI_{\text{init}} - EE - \rho_G \frac{dG}{dt}\Big), \\ \frac{dL}{dt} &= \frac{p}{\rho_L}\Big(TEI_{\text{init}} - EE - \rho_G \frac{dG}{dt}\Big), \end{aligned} \tag{17}$$

with $\rho_F = 9440.727$ kcals/kg (39.5 MJ/kg), $\rho_L = 1816.444$ kcals/kg (7.6 MJ/kg) are constants, and $p \equiv C/(C+F)$ a function of fat mass with $C = 10.4 \cdot \rho_L \rho_F^{-1}$. Total energy expenditure EE is given by:

$$EE = K + \gamma_F F + \gamma_L L + \delta BW + TEF + AT + \eta_L \frac{dL}{dt} + \eta_F \frac{dF}{dt}, \tag{18}$$

with $\gamma_F = 3.107075$ kcals/kg/d (13 kJ/kg/d), $\gamma_L = 21.98853$ kcals/kg/d (92 kJ/kg/d), $\eta_F = 179.2543$ kcals/kg (750 kJ/d), $\eta_L = 229.4455$ kcals/kg (960 kJ/kg) are physiological constants. K is determined by the initial energy balance condition:

$$K = RMR_{init} \cdot PAL - \gamma_L L_{init} - \gamma_F F_{init} - \delta BW_{init} \tag{19}$$

with RMR_{init} the initial resting metabolic rate (as estimated by (21)), PAL the physical activity level (assumed $PAL = 1.5$), $L_{init}, F_{init}, BW_{init}$ the initial lean, fat and body weight masses. The constant δ is determined defined as $\delta = RMR_{init}\Big((1 - \beta_{TEF}) \cdot PAL - 1\Big)/BW_{init}$ with $\beta_{TEF} = 0.1$. Furthermore, the thermal effect of feeding is defined as $TEF \equiv \beta_{TEF}\Delta TEI(t)$ with $\Delta TEI(t)$ as specified in (13). Finally, adaptative thermogenesis is given by the solution to the ODE system:

$$\begin{cases} \dfrac{dAT}{dt} & = \beta_{AT}\Delta TEI - AT, \\ AT_{init} & = \beta_{AT} \cdot PAL \cdot RMR_{init}. \end{cases} \tag{20}$$

We remark that for each individual, the initial resting metabolic rate RMR_{init} is described by the equations [10]:

$$RMR_{init} = \begin{cases} 9.99 \cdot BW_{init} + 625 \cdot H_{init} - 4.92 \cdot AGE_{init} + 5 & \text{if Sex = Male}, \\ 9.99 \cdot BW_{init} + 625 \cdot H_{init} - 4.92 \cdot \cdot AGE_{init} - 161 & \text{if Sex = Female}. \end{cases} \tag{21}$$

with H_{init}, AGE_{init} initial height and age respectively. Initial fat mass was obtained via the function:

$$F_{init} = \begin{cases} (1/100) \cdot BW_{init} \cdot \Big(0.14 \cdot AGE_{init} + 37.31 \times \ln(BW_{init}/H_{init}^2) - 103.94\Big) & \text{if Sex} = \text{Male}, \\ (1/100) \cdot BW_{init} \cdot \Big(0.14 \cdot AGE_{init} + 39.96 \cdot \ln(BW_{init}/H_{init}^2) - 102.01\Big) & \text{if Sex} = \text{Female}. \end{cases} \tag{22}$$

Additional information on the model can be found in [9, 11, 12, 13]

3.1 Individual implementation

For each individual i in the ENSANUT sample we estimated their energy intake change (13) as a function of time from their individual SSB consumption, $SSB_{\text{init}}^{(i)}$ (`sugar_ssb` in database) and their reported total energy intake $TEI_{init}^{(i)}$ (`kcaltot`). We used this quantity to obtain their carbohydrate intake change $CI^{(i)}(t) = \Delta TEI^{(i)}(t)/2$ and their carbohydrate intake at baseline $CI_b^{(i)} = TEI_{init}^{(i)}/2$.

The individual's resting metabolic rate $RMR_{init}^{(i)}$ was estimated from their initial body weight in kilograms $BW_{init}^{(i)}$ (`weight_baseline` in database), height in centimeters $H_{init}^{(i)}$ (`height` in database), age in years $AGE_{init}^{(i)}$ (`age` in database), and sex, $\text{Sex}^{(i)}$ (`sex` in database) following (21). Their initial fat mass $F_{init}^{(i)}$ was also estimated from $BW_{init}^{(i)}, H_{init}^{(i)}, AGE_{init}^{(i)}, \text{Sex}^{(i)}$ in accordance to (22). The individual's initial lean mass was obtained by setting $L_{init}^{(i)} = BW_{init}^{(i)} - \big(F_{init}^{(i)} + 3.7 \cdot G_{init} + ECF_{init}\big)$, with $G_{init} = 0.5$ and $ECF_{init} = 0$. Finally each individual's energy balance constant was estimated as $K^{(i)} = RMR_{init}^{(i)} \cdot PAL^{(i)} - \gamma_L L_{init}^{(i)} - \gamma_F F_{init}^{(i)} - \delta^{(i)} BW_{init}^{(i)}$ where $\delta^{(i)} = RMR_{init}^{(i)}\Big((1-\beta_{TEF}) \cdot PAL^{(i)} - 1\Big)/BW_{init}^{(i)}$. For all individuals we set a physical activity level of 1.5 ($PAL^{(i)} = 1.5$) which corresponds to "sedentarism" in accordance to [9].

For each individual, we estimated lean and fat masses, glycogen and extracellular fluid from the system of equations given by (15—20) using the parameters described above and setting $\Delta NA_{diet}^{(i)} = 0$.[2] To solve this system of differential equations, we used a 4th order Runge-Kutta algorithm (RK4) [14] with a stepsize $\Delta t = 1$. RK4 was programmed in `C++` for speed and connected to `R` via the `Rcpp` package.[15, 16].

The RK4 algorithm throws numerical estimates for each time t of each individual's extracellular fluid $ECF^{(i)}(t)$, glycogen $G^{(i)}(t)$, fat and lean masses $F^{(i)}(t), L^{(i)}(t)$. We estimated body weight for each individual adult in the ENSANUT sample as:

$$BW^{(i)}(t) = ECF^{(i)}(t) + 3.7 \cdot G^{(i)}(t) + F^{(i)}(t) + L^{(i)}(t)$$

where t stands for the number of days since the intervention. Each individual's BMI was estimated as:

$$BMI^{(i)}(t) = BW^{(i)}(t)/(H_{init}^{(i)})^2.$$

The previous model is completely programmed in the `bw` package in `R` [17]. Finally, we used the `survey` package [18, 19] to create summary statistics of $BW^{(i)}(t)$ and $BMI^{(i)}(t)$ (both in the overall adult population and in specific subpopulations by sex, SES, and age). For these estimates we accounted for the survey design as established in section 1.2.

This model has been written in pseudocode and is presented in Algorithm 1.

The different scenarios described in section 2.3 were implemented following the same algorithm by changing the formula for the reduction by year k in (13) and thus obtaining a different $\Delta TEI(t)$.

[2] Albeit we assumed no change in sodium intake $\Delta Na_{diet} = 0$, reducing sugar in SSB can have an effect on the recipe resulting in sodium intake changes.

Algorithm 1 Individual level weight change model

procedure WEIGHT CHANGE MODEL
Input:
n ▷ `Number of individuals in sample`
Years ▷ `Number of years to run the model for`
w_k ▷ `Survey weight for` k`-th individual` $(k = 1, 2, \ldots, n)$
$TEI_{\text{init}}^{(k)}$ ▷ k`-th individual's reported total energy intake` $(k = 1, 2, \ldots, n)$
$SSB_{\text{init}}^{(k)}$ ▷ k`-th individual's reported SSB consumption` $(k = 1, 2, \ldots, n)$
$BW_{\text{init}}^{(k)}$ ▷ k`-th individual's reported body weight` $(k = 1, 2, \ldots, n)$
$H_{\text{init}}^{(k)}$ ▷ k`-th individual's reported height` $(k = 1, 2, \ldots, n)$
$AGE_{\text{init}}^{(k)}$ ▷ k`-th individual's reported age` $(k = 1, 2, \ldots, n)$
$\text{Sex}^{(k)}$ ▷ k`-th individual's sex` $(k = 1, 2, \ldots, n)$
$PAL^{(k)}$ ▷ k`-th individual's physical activity level` $(k = 1, 2, \ldots, n)$
$\Delta NA_{diet}^{(k)}$ ▷ k`-th individual's change in sodium intake` $(k = 1, 2, \ldots, n)$
for i **in** 1 **to** n **do**
$\Delta TEI(t)^{(i)} \leftarrow TEI_{\text{init}}^{(i)} - \text{Reduction}(y_{\lceil t/365 \rceil}) \cdot SSB_{\text{init}}^{(i)}$
$CI^{(i)}(t) \leftarrow \Delta TEI^{(i)}(t)/2$
$CI_b^{(i)} \leftarrow TEI_{init}^{(i)}/2$
Calculate $RMR_{init}^{(i)}$ from $BW_{init}^{(i)}, H_{init}^{(i)}, AGE_{init}^{(i)}, \text{Sex}^{(i)}$ using (21).
Calculate $F_{init}^{(i)}$ from $BW_{init}^{(i)}, H_{init}^{(i)}, AGE_{init}^{(i)}, \text{Sex}^{(i)}$ using (22).
$L_{init}^{(i)} \leftarrow BW_{init}^{(i)} - \left(F_{init}^{(i)} + 3.7 \cdot G_{init} + ECF_{init}\right)$
$K^{(i)} \leftarrow RMR_{init}^{(i)} \cdot PAL^{(i)} - \gamma_L L_{init}^{(i)} - \gamma_F F_{init}^{(i)} - \delta BW_{init}^{(i)}$ as in (19)
$\delta^{(i)} \leftarrow RMR_{init}^{(i)}\Big((1 - \beta_{TEF}) \cdot PAL^{(i)} - 1\Big)/BW_{init}^{(i)}$
Runge Kutta 4 do
Calculate $AT^{(i)}$ from (20) using $RMR_{init}^{(i)}$, $\Delta TEI^{(i)}$ and $PAL^{(i)}$.
Calculate $ECF^{(i)}$ from (15) using $CI^{(i)}$, $CI_b^{(i)}$ and $\Delta NA_{diet}^{(i)}$.
Calculate $G^{(i)}$ using $CI^{(i)}, CI^{(i)}$ as in (16)
Calculate $F^{(i)}$ and $L^{(i)}$ as in (17) using $AT^{(i)}, K^{(i)}, TEI^{(i)}, G^{(i)}, PAL^{(i)}, \Delta TEI^{(i)}$.
end Runge Kutta 4
$BW^{(i)}(t) \leftarrow ECF^{(i)}(t) + 3.7 \cdot G^{(i)}(t) + F^{(i)}(t) + L^{(i)}(t)$
$BW_F^{(i)} \leftarrow BW^{(i)}(365 \cdot \text{Years})$
$BMI_F^{(i)} \leftarrow BW_F^{(i)}/(H_{init}^{(i)})^2$
$\Delta BW^{(i)} \leftarrow BW_{init}^{(i)} - BW_F^{(i)}$
end for
$\overline{\Delta BW}_{\text{Overall}} = \sum_{i=1}^{n} w_i \cdot \Delta BW^{(i)}$
for cat **in** [Males, Females, SES low, SES middle, SES high, Age 20-39, Age 40-59, Age 60+] **do**
$\overline{\Delta BW}_{\text{cat}} = \sum_{i=1}^{n} w_i \cdot \Delta BW^{(i)} \cdot \mathbb{I}_{\text{cat}}$
end for
end procedure

4 Sensitivity analysis

4.1 Different scenarios

The increasing and constant percent change scenarios from sections 2.3.2 and 2.3.3 were implemented as a sensitivity analysis. These resulted in different functional forms for $\Delta TEI(t)$ as different formulas for the reduction were used in (13); the remaining part of the estimation process (section 3) was the same.

4.2 Model under compensation assumptions

As different combinations of compensation and regulations result in different values of energy reduction ($\lambda \times 100\%$, following the notation of section 2.3) at year 10, we created a consumption-percent change matrix Λ whose entries correspond to the overall reduction associated to both, the % compensation and the % added sugar reduction (Table F). The rows of the matrix stand for % added sugar reduction whilst the columns for % compensation (both in multiples of 10). Hence for a reduction of 10% ($1 \times 10\%$) and compensation of 30% ($3 \times 10\%$) the entry $\Lambda_{1+1,3+1}$ of the matrix equals $1 \times \big(10 - 3\big)\% = 7\%$. In general, each entry of the matrix corresponds to a $\lambda \times 100\%$ reduction es given by $\Lambda_{i+1,j+1} = i \times \big(10 - j\big)\%$. Table F shows the reductions $\Lambda_{i+1,j+1} \times 100\%$ modelled for the sensitivity analysis.

% Added sugar reduction \ % Compensation	**0**	**10**	**20**	**30**	**40**	**50**	**60**	**70**	**80**	**90**	**100**
**0**	0	0	0	0	0	0	0	0	0	0	0
**10**	10	9	8	7	6	5	4	3	2	1	0
**20**	20	18	16	14	12	10	8	6	4	2	0
**30**	30	27	24	21	18	15	12	9	6	3	0
**40**	40	36	32	28	24	20	16	12	8	4	0
**50**	50	45	40	35	30	25	20	15	10	5	0
**60**	60	54	48	42	36	30	24	18	12	6	0
**70**	70	63	56	49	42	35	28	21	14	7	0
**80**	80	72	64	56	48	40	32	24	16	8	0
**90**	90	81	72	63	54	45	36	27	18	9	0
**100**	100	90	80	70	60	50	40	30	20	10	0

Table F: *Matrix Λ with percent reductions. For $i \times 10\%$ added sugar reduction and $j \times 10\%$ compensation the entry $\Lambda_{i+1,j+1}$ ($(i+1)$-th row, $(j+1)$-th column) denotes the corresponding % added sugar diminishment.*

Each entry $\lambda \equiv \Lambda_{i,j}$ of the matrix was applied to the main scenario (7) to obtain the k-th year reduction. The reductions resulting from (7) were then plugged into (13) and the weight change model (section 3) was applied. There results were associated to a weight reduction matrix W whose entries $W_{i+1,j+1}$ correspond to weight (kg) reduced after added sugar reduction to $i \times 10\%$ accounting for $j \times 10\%$ compensation. We represented the matrix graphically with the `ggplot2` package [20] using cell-shading as seen in figure 1 in the main article.

References

[1] Instituto Nacional de Salud Pública. Encuesta nacional de salud y nutrición. `http://ensanut.insp.mx/`, 2012. Online; accessed 24 May 2016.

[2] Ana Abreu and Rodrigo Zepeda-Tello. Dataset for regulating added sugars in sweetened beverages in mexico. `osf.io/vfcm8`, June 2018.

[3] Tania G Sánchez-Pimienta, Carolina Batis, Chessa K Lutter, and Juan A Rivera. Sugar-sweetened beverages are the main sources of added sugar intake in the mexican population. *The Journal of nutrition*, 146(9):1888S–1896S, 2016.

[4] Martín Romero-Martínez, Teresa Shamah-Levy, Aurora Franco-Núñez, Salvador Villalpando, Lucía Cuevas-Nasu, Juan Pablo Gutiérrez, and Juan Ángel Rivera-Dommarco. Encuesta nacional de salud y nutrición 2012: diseño y cobertura. *Salud publica de Mexico*, 55:S332–S340, 2013.

[5] R Core Team. *R: A Language and Environment for Statistical Computing.* R Foundation for Statistical Computing, Vienna, Austria, 2018.

[6] Thomas Lumley et al. Analysis of complex survey samples. *Journal of Statistical Software*, 9(1):1–19, 2004.

[7] StataCorp. Stata statistical software: Release 14. `https://www.stata.com/`.

[8] Y. Ma, F. J. He, Y. Yin, K. M. Hashem, and G. A. MacGregor. Gradual reduction of sugar in soft drinks without substitution as a strategy to reduce overweight, obesity, and type 2 diabetes: a modelling study. *Lancet Diabetes Endocrinol*, 4(2):105–114, Feb 2016.

[9] Carson C Chow and Kevin D Hall. The dynamics of human body weight change. *PLoS Comput Biol*, 4(3):e1000045, 2008.

[10] Mark D Mifflin, Sachiko T St Jeor, Lisa A Hill, Barbara J Scott, Sandra A Daugherty, and YO Koh. A new predictive equation for resting energy expenditure in healthy individuals. *The American journal of clinical nutrition*, 51(2):241–247, 1990.

[11] Kevin D Hall, Gary Sacks, Dhruva Chandramohan, Carson C Chow, Y Claire Wang, Steven L Gortmaker, and Boyd A Swinburn. Quantification of the effect of energy imbalance on bodyweight. *The Lancet*, 378(9793):826–837, 2011.

[12] Kevin D Hall. Predicting metabolic adaptation, body weight change, and energy intake in humans. *American Journal of Physiology-Endocrinology and Metabolism*, 298(3):E449–E466, 2010.

[13] Kevin D Hall and Peter N Jordan. Modeling weight-loss maintenance to help prevent body weight regain. *The American journal of clinical nutrition*, 88(6):1495–1503, 2008.

[14] Uri M Ascher and Chen Greif. *A first course on numerical methods.* SIAM, 2011.

[15] Dirk Eddelbuettel. *Seamless R and C++ Integration with Rcpp.* Springer, New York, 2013. ISBN 978-1-4614-6867-7.

[16] Dirk Eddelbuettel and Romain François. Rcpp: Seamless R and C++ integration. *Journal of Statistical Software*, 40(8):1–18, 2011.

[17] Dalia Camacho-García-Formentí and Rodrigo Zepeda-Tello. *bw: Dynamic Body Weight Models for Children and Adults*, 2018. R package version 1.0.0.

[18] Thomas Lumley. survey: analysis of complex survey samples, 2016. R package version 3.32.

[19] Thomas Lumley. Analysis of complex survey samples. *Journal of Statistical Software*, 9(1):1–19, 2004. R package verson 2.2.

[20] Hadley Wickham. *ggplot2: Elegant Graphics for Data Analysis.* Springer-Verlag New York, 2009.
