# Supplementary material for: Expected changes in obesity after reformulation to reduce added sugars in beverages: A modeling study
Source: PLoS Med. 2018 Oct 5;15(10):e1002664. doi: 10.1371/journal.pmed.1002664 (PMC6173390; doi:10.1371/journal.pmed.1002664)
Supplement: S1 Results — (DOCX) [file pmed.1002664.s002.docx]

**Supplementary results (S1 results) to**

**” Regulating added sugars in sweetened beverages could curb the obesity epidemic in Mexico: a modeling study”**

Authors: Ana Basto-Abreu, Ariela Braverman-Bronstein, Dalia Camacho-García-Formentí, Rodrigo Zepeda-Tello, Barry M. Popkin, Juan Rivera-Dommarco, Mauricio Hernández-Ávila, Tonatiuh Barrientos-Gutiérrez.

S1 results describe the impact of a reformulation program to reduce added sugars in SSBs considering different scenarios. Table A presents the impact of the reformulation to reduce sugar content on SSBs considering 0% compensation. Table B presents the expected impact of reformulating the sugar content of SSBs in people who reported drinking sugar-sweetened beverages in the last 24 hours (51.8%, representing 39.5 million people). This proportion underestimates the contribution of non-regular SSBs consumers; according to ENSANUT’s FFQ over 75% of the Mexican adult population consumes SSBs at least once a week.[1] Fig A presents different scenarios of gradually reducing sugar content in SSBs considering 43% compensation. Finally, Table C presents the impact on weight using different modeling approaches.

| Table A. Predicted reduction in added-sugar intake, body weight, BMI and obesity (pp, % baseline and prevented cases), from a 50% gradual reduction of added-sugars to SSBs, considering a 0% compensation after 12 years. | | | | | | |  |
| --- | --- | --- | --- | --- | --- | --- | --- |
|  | **Added sugars and energy intake* [kcal/day]  (95% CI)** | **Weight [kg]**  **(95% CI)** | **BMI [kg/m2]**  **(95% CI)** | **Change in prevalence Abs, pp**  **(95% CI)** | **Percent change in prevalence, % baseline, (95% CI)** | **Prevented cases with obesity, million**  **(95% CI)** | |
| Average | -50.6 (-54.5, -46.6) | -2.3 (-2.5, -2.1) | -0.9 (-1.0, -0.8) | -6.3 (-7.8 ,-4.8) | -20.3 (-24.6 ,-15.9) | -4.8 (-5.9 ,-3.6) | |
| Sex |  |  |  |  |  |  | |
| *Male* | -64.0 (-70.3, -57.7) | -2.7 (-3.0, -2.5) | -1.0 (-1.1, -0.9) | -6.4 (8.7 ,-4.2) | -26.7 (-34.7 ,-18.7) | -2.3 (3.0 ,-1.4) | |
| *Female* | -38.3 (-43.0, -33.7) | -1.9 (-2.1, -1.6) | -0.8 (-0.9, -0.7) | -6.1 (8.1 ,-4.1) | -16.4 (-21.4 ,-11.5) | -2.4 (2.3 ,-1.1) | |
| Age group | |  |  |  |  |  | |
| *20-39* | -65.4 (-72.1, -58.7) | -2.8 (-3.1, -2.5) | -1.1 (-1.2, -1.0) | -6.6 (-8.7 ,-4.4) | -25.9 (-33.4 ,-18.4) | -2.2 (-1.2 ,-0.6) | |
| *40-59* | -45.1 (-51.2, -39.1) | -2.2 (-2.5, -1.9) | -0.9 (-1.0, -0.7) | -8.0 (-11.0 ,-5.0) | -20.1 (-26.9 ,-13.3) | -2.2 (-2.5 ,-1.1) | |
| *60+* | -25.7 (-31.8, -19.7) | -1.2 (-1.5, -0.9) | -0.5 (-0.6, -0.4) | -2.1 (-3.2 ,-1.0) | -7.8 (-11.9 ,-3.6) | -0.3 (-0.7 ,-0.2) | |
| Socioeconomic status | |  |  |  |  |  | |
| *Low* | -42.2 (-48.6, -35.9) | -1.9 (-2.1, -1.6) | -0.7 (-0.8, -0.6) | -5.5 (-8.3 ,-2.7) | -20.5 (-29.4 ,-11.6) | -1.2 (-2.5 ,-0.8) | |
| *Middle* | -59.0 (-66.7, -51.3) | -2.6 (-3.0, -2.3) | -1.0 (-1.2, -0.9) | -6.9 (-9.4 ,-4.3) | -21.2 (-28.5 ,-13.9) | -1.6 (-3.4 ,-1.6) | |
| *High* | -50.4 (-57.0, -43.8) | -2.3 (-2.6, -2.0) | -0.9 (-1.0, -0.8) | -6.4 (-8.8 ,-4.0) | -19.4 (-26.0 ,-12.8) | -2.0 (-3.5 ,-1.6) | |

* Net energetic change assuming 43% compensation for sources other than added sugars

pp: percentage points to reflect the absolute difference

| Table B. Predicted reduction in added-sugar intake, energy intake, body weight, BMI and obesity (pp, % baseline and prevented cases) for regular SSBs consumers considering a 50% gradual reduction of added-sugars to SSBs and a 43% compensation, after 12 years. | | | | | | | |
| --- | --- | --- | --- | --- | --- | --- | --- |
|  | **Added sugars [kcal/day]**  **(95% CI)** | **Energy intake***  **[kcal/day]**  **(95% CI)** | **Weight [kg] (95% CI)** | **BMI [kg/m2] (95% CI)** | **Change in prevalence Abs, pp (95% CI)** | **Percent change in prevalence, % baseline, (95% CI)** | **Prevented cases with obesity, million (95% CI)** |
| Average | -97.7 (-103.3, -92.0) | -55.7 (-58.9, -52.5) | -2.5 (-2.7, -2.4) | -1.0 (-1.0, -0.9) | -7.5 (-9.8 ,-5.2) | -22.9 (-29.2 ,-16.6) | -3.0 (-3.9 ,-2.0) |
| Sex |  |  |  |  |  |  |  |
| *Male* | -112.4 (-120.6, -104.2) | -64.1 (-68.8, -59.4) | -2.8 (-3.0, -2.6) | -1.0 (-1.1, -0.9) | -7 (-10.2 ,-3.7) | -27.3 (-37.9 ,-16.7) | -1.3 (-1.9 ,-0.7) |
| *Female* | -81.5 (-88.9, -74.0) | -46.4 (-50.7, -42.2) | -2.3 (-2.5, -2.1) | -1.0 (-1.1, -0.9) | -8 (-11.4 ,-4.7) | -19.9 (-27.5 ,-12.3) | -1.7 (-2.3 ,-1.0) |
| Age group | |  |  |  |  |  |  |
| *20-39* | -106.7 (-115.3, -98.0) | -60.8 (-65.7, -55.9) | -2.6 (-2.9, -2.4) | -1.0 (-1.1, -0.9) | -7.7 (-10.8 ,-4.5) | -29.0 (-39 ,-19.0) | -1.3 (-1.9 ,-0.8) |
| *40-59* | -95.2 (-104.0, -86.4) | -54.3 (-59.3, -49.2) | -2.6 (-2.9, -2.4) | -1.0 (-1.1, -0.9) | -8.7 (-13.2 ,-4.3) | -18.9 (-27.8 ,-10) | -1.3 (-1.9 ,-0.6) |
| *60+* | -68.7 (-80.4, -57.1) | -39.2 (-45.8, -32.5) | -1.9 (-2.2, -1.6) | -0.8 (-0.9, -0.6) | -3.6 (-6.2 ,-0.9) | -15.9 (-26.7 ,-5.0) | -0.3 (-0.5 ,-0.1) |
| Socioeconomic status | |  |  |  |  |  |  |
| *Low* | -95.4 (-105.3, -85.5) | -54.4 (-60.0, -48.7) | -2.4 (-2.7, -2.2) | -1.0 (-1.1, -0.9) | -10.9 (-16.8 ,-5) | -35.6 (-50.3 ,-21.0) | -1.3 (-2.0 ,-0.6) |
| *Middle* | -105.8 (-115.7, -95.8) | -60.3 (-66.0, -54.6) | -2.7 (-3.0, -2.5) | -1.1 (-1.2, -1.0) | -5.6 (-8.5 ,-2.6) | -15.8 (-23.9 ,-7.7) | -0.7 (-1.0 ,-0.3) |
| *High* | -92.9 (-102.2, -83.6) | -52.9 (-58.2, -47.6) | -2.5 (-2.7, -2.2) | -0.9 (-1.0, -0.8) | -6.9 (10.1 ,-3.6) | -21.7 (-31.2 ,-12.2) | -1.1 (1.6 ,-0.6) |
| * Net energetic change assuming 43% compensation for sources other than added sugars | | | |  |  |  |  |
| pp: percentage points to reflect the absolute difference | | |  |  |  |  |  |

Fig. A. Three reduction schemes of gradually reducing added sugars in SSBs, over 12 years, considering 43% compensation.

| *Table C. Sensitivity analysis of sugar reduction on SSBs using different modeling approaches: comparative risk assessments and individual-based models*. | | | | | |  | |  |  |  |
| --- | --- | --- | --- | --- | --- | --- | --- | --- | --- | --- |
| **Study** | **Time frame** | **Allows for compensation** | **Modeling procedure** | **Parameter** | **Weight change (kg)** | |  |  |  |  |
| Hall, 2008 [2] | 12 years | Yes (43%) | Systems of ordinary differential equations based on time (12 y) that predicts individual body weight based on individual characteristics such as weight, height, energy intake and physical activity. | | -1.31 | |  |  |  |  |
|  | 12 years | No |  |  | -2.28 | |  |  |  |  |
| Briggs, 2017 [3] | 6 months | Yes, free feeding RCT | Comparative risk assessment based on a meta-analysis of 2 randomized controlled trials of SSBs consumption and body weight | Increase in weight of 0.09 kg per 100ml SSBs consumed | -0.12 | |  |  |  |  |
| Stern, 2017 [4] | 2 years | Yes, free feeding cohort study | Comparative risk assessment based on a cohort study in women in Mexico of SSBs consumption and body weight | Increase in weight of 1kg per 1 serving SSBs | -0.44 | |  |  |  |  |
| Christiansen, 2002 [5] | Long-term | Yes (43%) | Ordinary differential equation that predicts individual body weight based on changes in physical activity and energy intake. Assuming a steady state at baseline, the solution is simplified as:  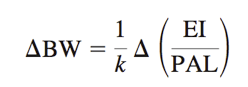where k=13.5 kcal/kg/day for men, k=11.6 kcal/kg/day for women, PAL-physical activity level, EI-energy intake. | | -1.52 | |  |  |  |  |
|  | Long-term | No |  |  | -2.66 | |  |  |  |  |

**References**

1. Barrientos-Gutierrez T, Zepeda-Tello R, Rodrigues ER, Colchero-Aragonés A, Rojas-Martínez R, Lazcano-Ponce E, et al. Expected population weight and diabetes impact of the 1-peso-per-litre tax to sugar sweetened beverages in Mexico. PLoS One. 2017;12: e0176336. doi:10.1371/journal.pone.0176336

2. Chow CC, Hall KD. The dynamics of human body weight change. PLoS Comput Biol. 2008;4: e1000045.

3. Briggs ADM, Mytton OT, Kehlbacher A, Tiffin R, Elhussein A, Rayner M, et al. Health impact assessment of the UK soft drinks industry levy: a comparative risk assessment modelling study. Lancet Public Heal. 2017;2: e15–e22. doi:10.1016/S2468-2667(16)30037-8

4. Stern D, Middaugh N, Rice MS, Laden F, López-Ridaura R, Rosner B, et al. Changes in sugar-sweetened soda consumption, weight, and waist circumference: 2-year cohort of Mexican women. Am J Public Health. 2017; doi:10.2105/AJPH.2017.304008

5. E. C, L. G. Prediction of body weight changes caused by changes in energy balance. Eur J Clin Invest. Wiley/Blackwell (10.1111); 2002;32: 826–830. doi:10.1046/j.1365-2362.2002.01036.x
